# 

**Published:** 1831

**Authors:** 


### BIBLIOGRAPHICAL RECORD;

OR,

*Works received for Review since last Quarter.*

1. A practical Treatise on Injuries of the Head. Small 8vo, pp. 121. Dublin, June 1831. Price 3s. 6d.

2. Pathological and practical Observations on Spinal Distortions, illustrating an improved Mode of Treatment without the Aid of Machinery. By F. P. B. Peckthorn, Esq. Octavo, pp. 85. London, June 1831.

3. Treatise on Cholera Morbus; the Method of Treatment, and the Means of Prevention. Written for the Emperor of

Russia's Prize: dedicated to His Imperial Majesty. By W. WHITE, of the Hon. East India Company's Service. Octavo, sewed, pp. 36. July 1831.

---

4. Du Procédé Operatoire a suivre dans l'Exploration des Organes par la Percussion Médiate, et Collection de Mémoires sur la Physiologie, la Pathologie, et le Diagnostic. Par P. A. PIORRY, M. D. Octavo, pp. 425. Paris and London, 1831.

☞ *Noticed in this Number, page* 488.

---

5. A Manual of Materia Medica and Pharmacy, comprising a concise Description of the Articles used in Medicine; with Observations on the proper Mode of combining and administering them; also the Formulæ for the officinal Preparations of the London, Edinburgh, Dublin, Parisian, American, and most of the continental Pharmacopœiæ, together with a Table of the Medicinal Plants. From the French of H. M. EDWARDS, M.D. and P. VAVASSEUR, M. D. Corrected and adapted to British Practice. By JOHN DAVIES, M.R.C.S. &c. One vol. 8vo. pp. 490. 1831.

☞ *This work, we believe, was published first in America—at least, it was translated into English there. Mr. Davies has performed his part with sufficient care, and the volume will be found very useful to two classes of readers—students and chemists.*

---

6. First Lines of the Practice of Midwifery: to which are added, Remarks on the Forensic Evidence requisite in Cases of Fœticide and Infanticide. By CHARLES SEVERN, Surgeon. Octavo, pp. 143. With Plates. London, 1831.

☞ *This is a very judicious, yet concentrated compilation, interspersed with original observations. It is creditable to the author, and will be found useful to the student and junior practitioner, when some trifling errors are corrected.*

---

Dyspepsy forestalled and resisted; or, Lectures on Diet, Regimen, and Employment, delivered to the Students of Amherst College, Spring Term, 1830, by EDWARD HITCHCOCK, Professor of Chemistry and Natural History. Second edit. enlarged, pp. 452. New York, 1831.

☞ *This is a valuable compilation. We shall notice some detached articles in our Periscope.*

---

8. History of the Epidemic Spasmodic Cholera of Russia; including a copious Account of the Disease which has prevailed in India, and which has travelled, under that name, from Asia into Europe. Illustrated by numerous official and other Documents, &c. By BISSET HAWKINS, M.D. &c. Octavo, pp. 306. 1831.

☞ *A very able and diligent compilation, not pretending to any original information.*

---

9. Essays on various Subjects of Medical Science. By DAVID HOSACK, M. D. F.R.S. &c. Octavo, pp. 473. New York, 1830.

---

10. An Account of Inventions and Improvements in Surgical Intruments, made by JOHN WEISS, 62, Strand; with a Selection of Cases wherein they have been successfully employed, and Testimonials of their Utility from eminent Surgeons. Illustrated by numerous Engravings. *Second Edit.* much enlarged, Price 15s. boards. Longman's, 1831.

☞ *The title sufficiently explains the nature of the volume. Independently of the* ARMAMENTUM CHIRURGICUM, *illustrated by plates and descriptions, the present edition contains a great number of curious cases detailed by individuals in letters or by publication.*

---

11. A Treatise on the Practice of Medicine. In two volumes, 8vo. By JOHN EBERLE, M.D. Professor of Materia Medica and Obstetrics, &c. Philadelphia, 1830.

☞ *This appears, on a hasty glance, to be one of the ablest and most original works of the kind which we have seen.*

---

12. Physiologie Medicale et Philosophique. Par ALM. LEPELLETIER DE LA SARTHE. 4 vols. 8vo.

☞ *The first volume only has yet been published, but the whole is to be completed in the course of the present year. It appears, from this first specimen, to be a work of merit, and the author unquestionably a man of talent and judgment.*

13. Cholera Morbus. A short and faithful Account of the History, Progress, Causes, Symptoms, and Treatment of the Indian and Russian Cholera, taken from various authentic Sources, &c. &c. By JOHN AUSTIN, Surgeon. Octavo, pp. 44. Sewed, price one shilling.

---

14. The Art of Cupping; being a brief History of the Operation, from its Origin to the present Time; its Utility; minute Rules for its Performance, &c. By GEORGE FREDERIC KNOX, Cupper at the Westminster Hospital, &c. Octavo, pp. 68. Highley, 1831.

---

15. Die Epidemische Cholera, &c. The Epidemic Cholera, or Morbus Emesi Catharsis; an Essay read before the Physiological Society of Leipsic, Dec. 1830. By MAURICE HOSPER, M. D. Professor, &c. in the University of Leipsic. Octavo.

☞ *This Essay is on the same plan as Dr. Hawkins' publication in this country. It is ably compiled, and interspersed with many ingenious remarks and speculations. But it only reaches to the latter end of the year* 1830, *and consequently falls short of the information contained in Dr. H's book.*

---

16. Practical Observations on Prolapsus of the Rectum. By FRED. SALMON, F. R. C. S. &c. Octavo, pp. 105, with plates. 1831.

---

17. Medico-Chirurgical Notes and illustrations. By R. FLETCHER, Esq. 4to, pp. 146, with plates. 1831.

☞ *Amply reviewed in our present number.*

---

18. A Manual of Medical Jurisprudence, compiled from the best medical and legal works: comprising an account of *first*, the ethics of the medical profession—*secondly*, the charters and statutes relating to the faculty—and *thirdly*, all medico-legal questions, with the latest decisions; being an analysis of a course of lectures on forensic medicine, &c. By MICHAEL RYAN, M. D. &c. Octavo, pp. 310. Renshaw and Rush, Sept. 1831.

---

19. An Account of a Contagious Fever which occurred among the Danish and American Prisoners of War at Chatham, in the years 1813, 1814. By Sir WILLIAM BURNETT, Knt. M.D. K.C.H. &c. Octavo, pp. 47. August 1831.

20. A Corn. Celsi Medicinæ, libri octo, ex Recensione Leonardi Targæ. Quibus accedunt tituli marginales perpetui, capitum librorumque; Annotationes Criticæ, &c. EDUARDUS MILLIGAN, M.D. &c. Editio Secunda, Auctior et Castigation. Octavo, pp. 639. Price 16 shillings. Edinburgh and London. Sept. 1831.

☞ *See Periscope.*

---

21. An inaugural Dissertation on the Congenital Malformations of the Heart. By JOHN PAGET, M. D. President of the Royal Medical Society. Octavo, pp. 54. Edinburgh, 1831.

---

22. Elements of Practical Midwifery; or, Companion to the Lying-in Room. By CHARLES WALLER, Consulting Accoucheur to the London and Southwark Midwifery Institution, &c. Duodecimo, pp. 199, with three plates. Second Edit. Sept. 1830, price 4s. 6d. Highley, Fleet Street.

☞ *The rapid sale of the first edition has induced the author to amend, and add to, the second edition, which is thus improved.*

---

23. Aur. Corn. Celsus on Medicine, in Eight Books, Latin and English. Translated from L. Targa's Edition, the words of the text being arranged in the order of construction. To which are prefixed, a Life of the Author, Tables of Weights, explanatory Notes, &c. designed to facilitate the Progress of Medical Students. By ALEX. LEE, A.M. Surgeon. In two volumes. Vol. I. 8vo. pp. 318. Cox, Borough, 1831.

☞ *See this number of Journal.*

---

*In the Press.*

Remarks on the subject of Lactation, &c. By Dr. MORTON.

---

THE LATE DR. GOOCH.

A most excellent likeness of this distinguished physician and amiable man has been drawn and engraved by the same artist—Mr. Linnell. To all who wish to possess themselves of a faithful portrait of Dr. Gooch, we conscientiously recommend this excellent engraving.

### INDEX.

A.

Abdomen, chronic tumour in the .. 498
Abdomen, tubercular disease of.... 466
Abdominal tumour, case of ........ 246
Abernethy, the late Mr. .......... 286
Abernethy, the late Mr. .......... 563
Abscess of the pelvis after lithotomy 367
Abscess of the tonsil, best mode of opening .......................... 384
Abscess of the cheek, from bad teeth 474
Abscess over the liver—curious case 274
Abstinence in disease of heart .... 491
Abstinence in diseases of lungs .... 492
Abstinence in diseases of bowels .. 492
Abstinence, virtues of ............ 571
Acute arteritis, supposed cases of .. 219
Aikin, Mr. on tartrite of iron and ammonia .......................... 454
Air, advantages of change of ...... 189
Alison, Dr. his outlines of physiology 75
Amputation, partial of the foot .... 124
Amputation of shoulder-joint...... 513
Amputation at hip-joint .......... 513
Amputation, fatal, for fungous tumour ............................ 522
Anatomical models, Mr. Schloss's.. 266
Anatomy in the 16th century...... 407
Anatomy first taught in Alexandria . 398
Anderson, Dr. on arsenic.......... 244
Andral, M. on cholera............. 208
Andral, M. on diseases of the liver . 85
Andral, M. on the blood .......... 337
Anecdote of Dr. Walker .......... 29
Aneurism by anastomosis......... 176
Angina pectoris, case of .......... 216
Annesley, Mr. on dysentery....... 102
Aorta, extensive disease of the .... 449
Aortic aneurism, bursting into the œsophagus ..................... 508
Apothecaries' Company's rules for lectures ......................... 145
Aquo-capsulitis, Mr. Mackenzie on . 69
Arabian physicians .............. 402
Arsenic, on its therapeutic properties 244
Arteries, torsion of .............. 177
Arthritis treated by acupuncture .. 468
Aural aneurism, successful operation 472
Axillary aneurism cured .......... 482
Axillary aneurism cured .......... 507

B.

Baron, Dr. his contributions to pathology ......................... 246
Baths of Liebenzell .............. 256
Beale, Mr. on distortions of the spine 418
Beatty, Dr. on difficult labours .... 91
Bennett, Mr. his last illness ...... 251
Bile, alterations of the........... 90
Biliary ducts, diseases of the ...... 89
Biliary calculi, deceptive symptoms 218
Blacklock, Mr. on inflation of the bowels ............................ 267
Bladder, case of cancer of the...... 453
Bland, M. on hooping-cough ...... 480
Bland, M. his physiology.......... 510
Bleedings, small, in hæmoptysis.... 146
Blood, M. Andral on the.......... 337
Blood, forces influencing it........ 337
Blood, alterations in the.......... 339
Blood, experiments on the ........ 342
Blood, influence of the nerves on the 344
Blood, inflammatory state of the.... 346
Blood, deleterious substances in the 346
Blood, state of, in chronic affections 348
Blood, state of, in dropsy, scurvy &c. 350
Blood, M. Piorry on the .......... 488
Bloody perspiration .............. 496
Board of Health on cholera........ 527
Boërhaave and Haller, notice of.... 410
Bougie, in stricture of œsophagus .. 387
Brain, abscess and ulceration of the 317
Brain, softening of the............ 322
Brain, effects of pressure on the. .. 326
Brain, sympathetic affections of the 296
Bright, Dr. his medical reports .... 289
British Madeira.................. 495
Brodie, Mr. on calculous disorders . 129
Bryce, Dr. his capital operations .. 513
Bulletins on his late Majesty ...... 200
Burne, Dr. on the pulse .......... 474
Burnett, Sir W. on a fever at Chatham ............................ 561
Bye-laws of the College of Surgeons 278

C.

Calculi, renal, Mr. Brodie on...... 136
Calculi in the bladder ............ 140
Calculi, composition of .......... 141
Calculi, encysted ................ 142
Calculi, symptoms of ............ 143
Calculi, with enlarged prostate .... 143
Calculi in the female bladder...... 257
Calculi, treatment of ............ 257
Calculi, dilatation of the urethra for 257
Calculi, solvents and injections for.. 259
Calculous disorders, Mr. Brodie on 129
Calculus extracted from the urethra 195
Cancer of rectum successfully extirpated ........................... 210
Cancer of the bladder, case of...... 453
Cancerous ulcerations of the face .. 196
Cantharides, poisoning by ........ 156
Carcinomatous ulceration, cases of, 536
Carotid tied for hemiplegia ........ 180
Catalepsy, case of................ 511
Cato's mode of reducing dislocations 399

Cava, inferior, obliteration of.......... 569
Cerebellum, injuries and diseases of 15
Cerebral inflammation, Dr. Bright 291
Chalybeate springs of Harrogate ... 356
Chancre, cases of.................. 225
Change of air, Dr. Ward on ......... 189
Cheyne, Dr. on hæmoptysis ......... 146
Chlorine, inhalation of, in phthisis.. 208
Cholera morbus, remarks on the ... 279
Cholera morbus, Dr. Drysen's report 280
Cholera morbus, Dr. Todd on...... 285
Cholera morbus, Mr. Herrmann ... 285
Cholera, M. Andral on............. 208
Cholera of India and Russia......... 447
Cholera, our treatment of ......... 448
Cholera, mode of preventing ...... 463
Cholera, papers on ............... 527
Cholera, post-mortem appearances in 528
Cholera, Drs. Russell and Barry on, 529
Cholera, symptoms of ............. 529
Choroiditis, Mr. Mackenzie on .... 63
Choroiditis, treatment of.......... 66
Circulating system, disease of the.. 449
Circulation in the lungs, Mr. Malden on the......................... 252
Clinique chirurgicale, M. Larrey's.. 1
Clinique of Boyer and Roux........ 521
Colchicum in cholera ............. 205
Cold, in poisoning by laudanum.... 327
Colleges of Surgeons and Physicians 162
College of Surgeons, bye-laws of the 278
Colic, inflation for ............... 157
Colon, spasm of the.............. 207
Colon, accumulation in the......... 498
Combe, Dr. on mental derangement 564
Contagion of cholera, M. Chervin on 500
Contagious fever at Chatham in 1813........................... 561
Contagiousness, acquired, of fever.. 562
Cooke, Mr. on the profession...... 526
Cooper, Mr. S. his case of aneurism, 508
Cornea and sclerotica, malformation 159
Corporations for promoting science 163
Corrigan, Dr. on spinal irritation.. 182
Corrigan, Dr. his theory looking foolish.......................... 220
Cranium, on suppuration in the.... 536
Croup, Dr. Goodlad on............. 241
Cupping, Mr. Knox on............. 471
Cusack, Dr. his report............. 21
Cyst communicating with stomach, 568

D.

Death from dread of operation .... 175
Delirium traumaticum, fatal case.. 272
Delirium tremens, cases of......... 294
Digitalis, morbid effects of......... 496
Disease, influence of, on the mind... 197
Disease, appearances of the tongue in 243
Dislocation, compound, of the os naviculare............................ 160
Distinction without separation .... 161
Distortions of the spine, Mr. Beale on 418
Duffin, Dr. his new pessary ....... 281
Dysentery, Mr. Annesley on ....... 102
Dysentery, acute ................ 102
Dysentery, hepatic ............... 105
Dysentery, post-mortem appearances 107
Dysentery, treatment of ........... 111

E.

Egg, Mr. on trusses............... 516
Enlarged prostate pierced for retention............................. 191
Epilepsy cured by iodine .......... 494
Epps, Dr. his life of Dr. Walker ... 26
Ergot of rye in hæmorrhages...... 171
Ergot of rye in leucorrhœa ........ 520
Erysipelas, contagiousness of? ..... 298
Erysipelas, punctures in .......... 300
Erysipelas, extending into the skull 302
Erysipelas, erratic, M. Rennes on .. 493
Essays and orations, by Sir H. Halford................................ 358
Excision of diseased joints, Mr. Syme 113
Excision of joints, method of performing.......................... 116
Excision of shoulder-joint ......... 117
Excision of elbow-joint ........... 119
Excision of wrist-joint............ 120
Excision of hip and knee-joints .... 121
Excision of ankle joint............ 123
Excision of scirrhous rectum ...... 159
Excision of the cervix uteri, *successful* ............................ 278
Exhaustion, death from, after lithotomy .............................. 263
Extra-uterine fœtation, case of..... 187
Eye, injuries of the .............. 20
Eye, Mr. Mackenzie on its diseases 52

F.

Facial neuralgia, case of.......... 511
Failures in lithotomy, Mr. Fletcher 365
Fletcher, Mr. his notes and illustrations ............................ 364
Fletcher, Mr. on prolapsus ani ..... 483
Fœtus, sanguification of the ....... 48
Fractured bones, re-union of....... 193
Fungoid disease, extensive, case of.. 304
Fungous tumour of radius, case of... 522
Fungus hæmatodes said to be cured 155

G.

Galen, historic notice of........... 400
Galvanism and nervous influence ... 83
Genitals in women, inflammation of 414
Genitals, ulcers of the ............ 416
Glans penis, inflammation of the .... 413
Glossitis, idiopathic, cases of....... 154
Glottis, Mr. Fletcher on spasm of the 378
Gonorrhœa and its consequences ... 221
Gonorrhœa, combined with syphilis 232
Gonorrhœal ophthalmia and rheumatism ............................ 224
Goodlad, Dr. on croup.............. 241
Gout, fatal case of................ 520
Graves, Dr. on the effects of posture 152

Green, Mr. on distinction without separation ... 161

Green, Mr. his plan of reform ... 168

Gun-shot wound of scalp ... 154

H.

Hæmorrhage in lithotomy ... 375
Hæmorrhages, ergot of rye in ... 171
Hæmoptysis, small bleedings in ... 146
Halford, Sir H. on the mental effects of disease ... 197
Halford, Sir H. his essays ... 358
Harrogate, Dr. Hunter on waters of 354
Harty, Dr. on polypi of the heart ... 96
Head, M. Larrey on injuries of the ... 7
Head, on injuries of the ... 532
Head, cases of injury of ... 505
Heart, abstinence in diseases of ... 491
Heart, motions and sounds of the ... 220
Heart, case of ulceration of ... 566
Hemiplegia, ligature of carotid for 180
Hernia, rapidly fatal case of ... 153
Hernia—Mr. Egg's trusses ... 516
Hip-joint, amputation at the ... 513
Hippocrates, notices respecting ... 395
History of medicine, Mr. Moir's ... 394
History of medicine, Mr. Hamilton's 394
Hitchcock, Prof. on dyspepsia ... 571
Holland, Dr. on the spleen, &c. ... 47
Hoo Loo, operation on ... 150
Hooping-cough, M. Bland on ... 480
Hospital gangrene, M. Larrey on ... 4
Hospital physicians, remarks on ... 289
Howship, Mr. his case of chronic tumour ... 498
Hyaloid membrane, inflammation of 73
Hydatids of the uterus ... 25
Hydrocele and hæmatocele cured ... 522
Hydrocephalic water, analysis of ... 316
Hydrocephalus, Dr. Bright on ... 306
Hydrocephalus, treatment of ... 312
Hydrocephalus, chronic, case of ... 314
Hydrocephalus, tapping for ... 317
Hydrophobia, M. Larrey on ... 2
Hydrothorax, paracentesis thoracis 149

I.

Imperforate anus, case of ... 268
Indigestion, Mr. Mayo on ... 41
Indo-Russian cholera ... 447
Inflammation of uterus, Dr. Lee on 425
Inflammation of the lens ... 71
Inflation for colic ... 157
Inflation of the bowels, in ileus, &c. 267
Injection, successful, of ovarian tumour ... 501
Injuries of head, report on ... 505
Injuries of the head, treatise on ... 532
Injury of the lungs—recovery ... 270
Injury of the spine—partial paralysis 274
Insanity and paralysis—dissection ... 186
Insanity, Sir H. Halford on ... 361
Institutions, medical, of this country 162
Instrument for lateral curvature ... 420
Intermittent tetanus ... 570
Intermittent affections, cases of ... 238
Iodine in cutaneous diseases ... 208
Iodine for epilepsy ... 494
Iris, absorption of the ... 158
Iritis, Mr. Mackenzie on ... 53
Iritis, rheumatic ... 56
Iritis, pseudo-syphilitic ... 59
Iritis, scrofulous ... 60
Iritis, arthritic ... 60

J.

Joints, diseased, excision of ... 113

K.

Key, Mr. his operation on Hoo Loo 150

Kidney ruptured, case of ... 281

L.

Labours, difficult, use of instruments in ... 91
Larrey, M. his clinique chirugicale 1
Larrey's mode of tapping the pericardium ... 514
Laryngitis—tracheotomy ... 464
Lateral curvature, instruments in ... 419
Lecturers, Apothecaries' Company's rules for ... 145
Lee, Dr. on uterine inflammation ... 425
Leucorrhœa, ergot of rye in ... 520
Life and organization ... 77
Lithontrity, remarks on ... 266
Lithotomy, Mr. Brodie on the operation of ... 259
Lithotomy, Mr. Brodie's method ... 261
Lithotomy, after treatment ... 262
Lithotomy, on death from ... 262
Lithotomy, high operation ... 264
Lithotomy in the female ... 265
Lithotomy, cases of failure in ... 365
Lithotomy, immediate death from ... 368
Lithotomy in the 15th century ... 405
Lithotomy in the female, various modes ... 515
Lithotomy, for the extraction of a catheter ... 522
Liver, Andral on diseases of the ... 35
Liver, cancer of the ... 39
Liver, case of rupture of the ... 269
Liver, abscess over the ... 274
London University, vile proceedings at the ... 330
Lower jaw, removal of part of ... 540
Lungs, on the circulation in the ... 252
Lungs, injury and inflammation of 270

M.

Mackenzie, Mr. on iritis ... 53

Mackenzie, Mr. on strabismus ... 523

Malden, Mr. on the pulmonary circulation ... 252

Mayo, Dr. on temperament ... 35

Medical zoology and mineralogy ... 126

Medical Provident Institution ... 453

Medicine, histories of ... 394

Medico-Chirurgical Notes. By Mr. Fletcher.......................... 364
Membranous inflammation, cause of death in.............................. 477
Memoires sur la physiologie ...... 488
Mental derangement with dissection, 217
Mental derangement, Dr. Combe on 564
Mercury in Indian diseases ........ 196
Mercury in a case of sloughing ulcer, 543
Mind, influence of disease on....... 197
Mineral waters of Harrogate, Dr. Hunter on.......................... 354
Monomania—phrenology.......... 172
Morbid anatomy, vade-mecum of .. 422
Muscular contraction, its causes ... 77
Mus musculus, comparative anatomy of.......................... 572

N.

Nævus, cases of .................. 176
Nævus, Dr. Marshall Hall's treatment of.......................... 177
Neuralgia of the heart, Dr. Elliotson on.......................... 212
Neuralgic rheumatism, case of .... 216
Nervous influence, electricity only.. 18
Nitric acid, dilute, injected into the bladder.......................... 259
Nostalgia, Mr. Larrey on.......... 18

O.

Obliteration of inferior cava ...... 569
Œsophagus, Mr. Fletcher on Strictures of.......................... 387
Œsophagus, diseases of the........ 423
Operation for aural aneurism...... 472
Ophthalmiæ, traumatic .......... 73
Ophthalmiæ, compound .......... 74
Ophthalmiæ, intermittent ........ 74
O'Shaughnessy, Dr. on poisoned confectionery.......................... 201
Ovarian tumour injected.......... 501

P.

Pancreas, inflammation of ........ 502
Paracentesis thoracis, for hydrothorax.......................... 149
Paralysis of lower extremities, case of.......................... 519
Paraphymosis, cases of............ 236
Pathological waltz, Dr. Badham's .. 277
Pattison, Professor, and London University.......................... 330
Periostitis, remarks on............ 567
Peritonitis from ruptured liver..... 269
Peritonitis from lithotomy ........ 371
Peritonitis, Dr. Lee on .......... 427
Pessary, Dr. Duffin's new ........ 281
Pharynx, false passage through the, 391
Philosophical physiology, M. Bland's 510
Phlebitis, idiopathic, case of ...... 240
Phrenology, Dr. Elliotson's belief in 173
Phthisis, inhalation of chlorine in.. 208
Phthisis cured by turtle .......... 284
Phthisis of spinners, Dr. Kay on .. 477
Phymosis, cases of.............. 233
Physician, a portion of his duty.... 199
Physiology of the spleen, &c...... 47
Physiology, Alison's outlines of .... 75
Piorry sur la physiologie.......... 488
Pleuritis and pneumo-thorax, case of, 212
Poisoned confectionary ............ 201
Polypi of the heart as a disease.... 96
Pomponius Atticus, cases of ...... 572
Population of Naples .............. 520
Practical men, their errors........ 36
Professor Bennett's last illness .... 251
Prolapsus ani, in grown persons.... 483
Prolapsus ani, cases of ............ 484
Prolapsus ani, operation for ...... 485
Prolapsus ani, with stricture of rectum.......................... 486
Psoas muscle, curious tumour attached to.......................... 157
Puerperal convulsions ............ 22
Puerperal fever, insidious form .... 518
Pulmonary abscess over the clavicle, 566
Pulse, effects of posture on the .... 152
Pulse, Dr. Burne on the.......... 474
Purulent discharge from the ear; cases.......................... 303

R.

Ramadge, Dr. and St. John Long .. 178
Ramollissement of the brain...... 322
Rectum, case of ulcer of the ...... 207
Rectum, scirrhus of the, extirpated, 210
Rectum, scirrhous, excision of .... 159
Red colour for confectionery analyzed.......................... 204
Renal calculi, Mr. Brodie on ...... 136
Renal calculi, descent of.......... 137
Reports of medical cases, Dr. Bright's 289
Republican freedom.............. 31
Retention of urine, with enlarged prostate.......................... 191
Retinitis, Mr. Mackenzie on ...... 66
Reunion of fractured bones........ 193
Roger Bacon, historic notice of.... 404
Roman air, curious effects of...... 282
Ryan, Dr. and his pupils.......... 563

S.

Sand, red, in the urine .......... 131
Sand, white, in the urine.......... 134
Scalp, on wounds of the .......... 532
Scalp, on contusions of the........ 533
Scalp, on erysipelas of the ........ 533
Scalp, gun-shot wound of the ...... 154
Scents in Rome .................. 282
Sciatica treated by acupuncture.... 468
Scirrhous rectum, excision of...... 159
Scotland Medical Institution ...... 453
Separation without dissention ...... 526
Sexual diseases, Mr. Thorn on.... 412
Shoulder-joint, amputation of...... 513
Sloughing ulcer of nose, case of.... 543
Sloughing chancres, cases of ...... 229
Sounding for stone, death from.... 370

Sounding for stone, inflamed bladder from.... 375
Spasm of the colon, Mr. Lyon on .. 207
Spasm of the glottis, cases of ...... 379
Spasm of the glottis, Mr. Fletcher on .... 378
Spasm of the glottis, treatment of.. 384
Spasmodic cholera, prevention of.. 463
Spectral illusions of a medical gentleman .... 205
Spine, Mr. Beale on distortions of.. 418
Spinal irritation, Mr. Whatton on.. 458
Spinal irritation, symptoms of .... 460
Spinal irritation, treatment of .... 462
Spinal irritation, case of .... 565
Spinal irritation, Dr. Corrigan on.. 182
Spinner's phthisis, Dr. Kay on .... 477
Spleen, physiology of the.... 48
Stephenson, Dr. his medical zoology 126
St. John Long and Dr. Ramadge ... 178
Stomach, cyst communicating with, 568
Strabismus, Mr. Mackenzie on .... 523
Strabismus, causes of .... 523
Strabismus, treatment of.... 524
Strangulated hernia rapidly fatal .. 153
Strictures of the œsophagus, cases of.... 387
Subclavian, successful ligature of .. 482
Subclavian tied with success ...... 507
Suffocation from ulcer of the throat, 381
Superficial sores on the genitals.... 235
Surgeons, unpopularity of the College of .... 164
Surgical anatomy, Mr. Burt's plates of.... 471
Surgical practitioners—their claims, 166
Syme, Mr. on excision of diseased joints .... 113
Sym, Dr. on tetanus.... 497
Syphilis, primary, cases of .... 225
Syphilis, its first appearance in England .... 405
T.
Tapping the pericardium, M. Larrey's mode .... 514
Tarsus and metatarsus, caries of .. 124
Tartrite of iron and ammonia...... 454
Temperament, Dr. Mayo on ...... 35
Temperament, the bilious ........ 37
Temperament, the nervous........ 38
Temperament, the sanguine ...... 39
Temperament, the phlegmatic .... 40
Test for contagiousness of cholera.. 500
Testis, inflammation of the ....... 223
Tetanus, remarks on .... 497
Tetanus, spontaneous .... 569
Tetanus, intermittent.... 570
The dead lion and living ass ...... 563
Thorn, Mr. on sexual diseases .... 412
Tic douloureux, Sir H. Halford on.. 358
Tight lacing, dreadful effects of.... 421
Tobacco, as an application in gout, 488
Tongue, appearance of, in disease.. 243
Tooth-ache, certain remedy for.... 457
Trachea, wound of the .... 238
Tracheotomy, successful.... 573
Tracheotomy for laryngitis.... 464
Traumatic delirium, fatal case of.. 272
Traumatic tetanus, cases of.... 174
Traumatic tetanus cured.... 215
Tube, respiration carried on through 573
Tubercular disease of the abdomen, 466
Tumours of the abdomen, cases of, 246
Turn of life, state at the.... 198
Tympanitis of the uterus .... 512
U.
Ulcer of the rectum, operated on .. 207
Ulceration, carcinomatous, cases of, 536
Ulceration of heart, case of.... 566
University of London and Professor Pattison .... 330
Urethra forceps, Mr. Brodie on the, 258
Urethra, female, dilatation of the.. 265
Urine, on sand in the .... 131
Uterine hæmorrhage .... 26
Uterine inflammation, Dr. Lee on.. 425
Uterine appendages, inflammation of 429
Uterine phlebitis .... 437
Uterine inflammation, causes of.... 440
Uterine inflammation, treatment of, 445
Uterus, inflammation of its tissue.. 432
Uterus, softening of the .... 432
Uterus, inflammation of its absorbents .... 435
Uterus, tympanitis of .... 512
V.
Vade-mecum of morbid anatomy .. 422
Venereal diseases, report on ...... 221
Venous absorption, Dr. Alison on .. 80
Vesico-vaginal fistula cured by suture.... 160
Vomica opening over the clavicle .. 566
W.
Wagstaff, Mr. on extra-uterine fœtation .... 187
Walker, Dr. his life, by Epps.... 26
Wall, Mr. his secret nostrum.... 537
Weatherill, Dr. his *successful* case.. 278
Wellesley Institution, report of the, 21
Whatton, Mr. on spinal irritation.. 458
Wound of the chest and liver, case of.... 211
Wound of the trachea—aerial fistula 238
Y.
Yellow colour of confectionery analyzed .... 202
Z.
Zoology, medical, Dr. Stephenson's, 126

### INDIGESTION—CHANGE OF AIR.

**AN ESSAY on INDIGESTION, or MORBID SENSIBILITY of the STOMACH and BOWELS,** as the proximate Cause, or characteristic Condition of Dyspepsia, Nervous Irritability, Mental Despondency, Hypochondriacism, and many other Ailments, with an improved Method of Treatment, Medicinal and Dietetic.

By JAMES JOHNSON, M.D. Physician Extraordinary to the KING. Seventh edit. enlarged, price 6s. 6d. boards.

*Just Published, by the same Author, price 8s. 6d. in boards,*

**CHANGE of AIR, or the PURSUIT of HEALTH**; being Autumnal Excursions through France, Switzerland, Italy, &c.; with Observations and Reflections on the Moral, Physical, and Medicinal Influence of Travelling Exercise, Change of Scene, Foreign Skies, and Mental Recreation, in Sickness and in Health.

Published by S. Highley, 32, Fleet-street.

☞ This last Work is already re-printed in New York by Mr. Wood, the republisher of the Medico-Chirurgical Review.

### CRITICAL NOTICES.

"Of all the popular Tours, of which British literature has recently been so prolific, this is immeasurably the best. To attempt an analysis of a work embracing such a treasure of anecdote and instruction, would be an idle task. There is no class of general readers which may not derive pleasure and profit from the perusal of this volume."—BALLOT.

"Dr. JOHNSON is a vigorous and independent thinker, while his opinions are slightly tinctured with cynicism, which gives them an agreeable relish. His style is clear, bold, and expressive; so that when he least aims at effect, he leaves a more vivid impression on us of the object of his reflections, than others would by an elaborate description."—MORNING HERALD.

"The author, out of his abundant stock of knowledge and reflection, has constructed a volume with which all classes will be pleased."—ATLAS.

"The medical portion embraces numerous remarks which we would recommend all invalids and their medical advisers to peruse, before they decide upon the dangerous experiment of foreign travel."—SPECTATOR.

"In his descriptions of the different countries he passed through, and the many objects of curiosity that attracted his notice, Dr. JOHNSON has preserved a freshness and originality, that reflect high credit upon his talent, as a writer and acute observer."—LONDON MED & PHYS. JOURN.

"The present publication is the most entertaining and edifying that has issued from the press for many years."—GAZETTE OF HEALTH.

"This work is so spirited, so full of sound moral reflection—so correct, and so impartial, that we scarcely know where to find its equal. It is a classical and philosophical tour. It is impossible to dip into any part of it, without having the attention rivetted and the fancy pleased."—LONDON MED. AND SURG. JOURNAL.

"Dr. JOHNSON has travelled with the spirit of a philosopher, and has thereby given an interest to his volume, which is not to be found in the dry journals of such as merely describe what they have seen, without being able to accomplish any thing beyond mere detail, unrelieved by reflections which will sometimes impart a charm and a freshness to the most hackneyed narrations. But the mental improvement to be derived from visiting the classic scenes through which the author of the book before us has travelled, is not obtained by merely looking upon the remains they present, and being able to describe their situation. It is in the sentiments they inspire that the mind becomes elevated; and to those who have not had the opportunity of seeing the places alluded to, Dr. Johnson's book will give the benefit of the reflections they are calculated to excite, for which alone they are objects of value, whatever may be their interest to posterity. It is not by the description of how many arches of the Coliseum remain complete, or how many pillars are yet standing in the Roman Forum, that the traveller in Italy can benefit his countrymen at home—nor by glowing descriptions of the beauties of Nature through which he may chance to have passed. He is of no advantage to society, unless the resources of his own genius enable him to find—

'Sermons in stones—books in the running brooks,
And good in every thing.'" LITERARY BEACON.

"We are greatly pleased with Dr. JOHNSON for the bold, fearless, uncompromising spirit in which he exposes the moral baseness, the vileness, the filthiness of the ancient, as well as of the modern Romans. It is true, he is sometimes a little too broad in his allusions, exposures, and sweeping censures; but for this he must stand excused, in the consideration that he had disgusting subjects to deal with, and in the consequent necessity of using plain language—of calling things by their right names—that he might be thoroughly understood, and that his denunciations might have due weight. An important service would have been rendered to the cause of truth, and to the interests of society, had some of our thousand and one travellers taken up the subject of the ancient and the modern Greeks in the same honest strain.

"There is much that is eminently curious and striking in this volume to the invalid, the tourist, the moralist, the philosopher:—and on the subject of health in particular, it deserves to be not only consulted, but studied, by every person, sound or unsound, previously to the undertaking of a journey to Italy."—LA BELLE ASSEMBLEE.

"Dr. JOHNSON is very far beyond an ordinary tourist: he travels for health or for relaxation, and gives, with the tact and the precision of his profession, the results of his own observations upon the physical effects of travelling. He looks with the eye of a philosopher, and something approaching to scorn, at the rage with which every thing is overdone, from ambition, pride, vanity, and fashion—the result, as it is, being loss of health and vigour. The reader will not be wearied with unimportant matters. The Doctor glances at every place, without any bother as to how he got there—what he ate—or where he slept on the road. In Rome, Naples, and Pompeii, he is full of historical recollections. The book is very superior—the author is a man of real intelligence—of considerable reading, and he brings it to bear occasionally with great felicity. There is sound knowledge at the bottom, and much that is well fitted to correct misconception and prejudice."—MONTHLY MAGAZINE, SEPT. 1831.

